# Role of Chemistry and Crystal Structure on the Electronic Defect States in Cs-Based Halide Perovskites

**DOI:** 10.3390/ma14041032

**Published:** 2021-02-22

**Authors:** Anirban Naskar, Rabi Khanal, Samrat Choudhury

**Affiliations:** Materials Science and Engineering Program, University of Idaho, Moscow, ID 83844, USA; nask9705@vandals.uidaho.edu (A.N.); rkhanal@uidaho.edu (R.K.)

**Keywords:** perovskite, electronic structure, defect properties

## Abstract

The electronic structure of a series perovskites ABX_3_ (A = Cs; B = Ca, Sr, and Ba; X = F, Cl, Br, and I) in the presence and absence of antisite defect X_B_ were systematically investigated based on density-functional-theory calculations. Both cubic and orthorhombic perovskites were considered. It was observed that for certain perovskite compositions and crystal structure, presence of antisite point defect leads to the formation of electronic defect state(s) within the band gap. We showed that both the type of electronic defect states and their individual energy level location within the bandgap can be predicted based on easily available intrinsic properties of the constituent elements, such as the bond-dissociation energy of the B–X and X–X bond, the X–X covalent bond length, and the atomic size of halide (X) as well as structural characteristic such as B–X–B bond angle. Overall, this work provides a science-based generic principle to design the electronic states within the band structure in Cs-based perovskites in presence of point defects such as antisite defect.

## 1. Introduction

ABX_3_ (A = monovalent organic or inorganic cation, B = bivalent metal, and X = halide) type perovskites have received much attention as candidate materials for various electronic and opto-electronic applications ranging from solar cells to light-emitting diodes [1,2,3]. The chemistry and crystal structure of these perovskites determine the band structure needed for the application. For example, the chemical bonding between the metal (B) and the halogen (X) atoms are linked to the valance band maxima (VBM) and conduction band minima (CBM) with anti-bonding X p and non-bonding B p states having major contributions at VBM and CBM, respectively [4]. The extent and nature of such orbital overlap is linked to intrinsic properties of the B and X atoms, such as the atomic radius [5] and the electronegativity (EN) differences between B and X [5,6]. The higher EN difference between B and X reportedly causes the widening of bandgap [7,8]. In an ionic bond, where the electronegativity difference between metal and halogen is high, the electronic charge cloud is less dispersed along the bond and localized near the nuclei, limiting the overlap between atomic orbitals and resulting in a large bandgap in ionic perovskite. Further, the size of the atom significantly influences the atomic orbital overlap in a bond. For example, in a covalent bond involving smaller sized atoms, the nucleus strongly pulls the electron cloud making it unable to form a covalent bond, and instead, forms an ionic bond with the other atoms. Further, atomic size approximates the energy of the orbitals that are participating in the bonding that leads to the band structure. Hence, the VBM and CBM positions vary systematically based on the atomic size. The effect of atomic size and electronegativity on the electronic structure of halide perovskites can be well understood from the bandgaps of MAPbX_3_ (X = I, Br, and Cl), a widely studied perovskite system used mainly for photovoltaic applications. The experimental bandgap of MAPbX_3_ (X = I, Br, and Cl) changes systematically from 1.55 eV [9], 2.00 eV [10], and 2.88 eV [11] with decreasing atomic size and increasing electronegativity from I, Br to Cl, respectively.

In addition to the atomic size and electronegativity, arrangement of atoms within a lattice can also significantly alter the positions of the VBM and CBM, and the band structure in general. In a typical halide perovskite structure, B metal is bonded with the neighboring six halides forming a [BX_6_]^4−^ octahedral, and the relatively larger A cation occupies the volume between two corner-sharing octahedra [12]. Based on the atomic arrangement, halide perovskites are mainly found in three temperature-dependent crystal structures: cubic, tetragonal, and orthorhombic. For instance, all MAPbI_3_ show a high-temperature cubic phase (α) followed by a tetragonal phase (β) at intermediate temperatures, and an orthorhombic phase (γ) at low temperatures [13]. These crystal structures in ABX_3_ perovskites differ in terms of their symmetry, or more precisely, on the lattice parameter and B-X–B bond angle in the [BX_6_] octahedra [12]. In the cubic structure, the [BX_6_] octahedra is oriented in such a way that all the B–X–B bond angles are 180°, whereas, in the tetragonal and orthorhombic phases, the octahedra is tilted with B–X–B bond angles less than 180°. A vast number of studies have claimed that a distortion of the [BX_6_] octahedra or B–X–B bond angles below 180° can change the bandgap in perovskites [14,15]. The deviation of the B–X–B bond angles from 180° in the ideal cubic structure reduces the antibonding interaction between the B and the X atoms, which lowers the energy of the VBM and increases the bandgap [15]. Xiao and co-workers reported that for CsPbI_3_, as the Pb–I–Pb bond angle (180°) in the cubic structure is reduced to 136.4° in the distorted structure, the bandgap increases to 1.66 eV from 1.48 eV [15].

Point defects, such as cation or halide vacancy, the interstitial and antisite defects produced during the synthesis of perovskite compounds, also affect the band structure. Frequently, these point defects introduce additional electronic states within the band structure which can affect the transfer of charge carriers across the bands. For example, cation and halide vacancy can form a state near the VBM and CBM (shallow state), whereas the antisite defect, which is formed usually at high halide concentrations, produces mid-gap localized states (trap state) [16,17]. In particular, both experimental and computational reports have linked the formation of localized electronic states to antisite point defect in lead halide perovskites [18,19,20].

In this paper, we performed density functional theory (DFT) calculations to systemically investigate the electronic structure of a series of theoretically and experimentally observed perovskites ABX_3_ (A = Cs; B = Ca, Sr, and Ba; X = F, Cl, Br, and I) in the presence and absence of antisite defect X_B_ (where one B atom is replaced by one X atom). It is notable that previous electronic structure calculations [21,22] on the Cs-based halide perovskites were mostly focused either on the defect formation energy or elemental contribution on the band structure. In this manuscript, we aim to establish a fundamental link between features of the perovskite electronic structure, such as band gap and the position of the localized electronic states w.r.t band edges, with the intrinsic atomic properties and crystal structure. An understanding of these relationships will help predict electronic band structure features for an arbitrary B and X combination based on easily available intrinsic features, such as EN, bond dissociation energy (BDE), and covalent radius of the B and X atoms. We restrict our study to the *s*-block perovskites (B = Ca, Sr, and Ba) as an alternative to Pb-based perovskites, which exhibit negative environmental impacts [23]. It is notable that both Pb-based and *s*-block perovskites exhibit similar elemental contribution toward band structure i.e., the valence and conduction band edges are formed by X and the B atoms, respectively [24,25].

## 2. Computation Details

Electronic structure calculations of ABX_3_ (A = Cs; B = Ca, Sr, and Ba; X = F, Cl, Br, and I) with and without point defect were performed using the Vienna *ab-initio* Simulation Package (VASP) [26]. It is notable that among the 12 possible chemical combinations of B and X, CsCaBr_3_ [27], CsCaF_3_ [28], and CsSrF_3_ [29] were experimentally observed in the cubic phase. Initial structures for other compositions are obtained from previous computational studies [30,31] and the materials project database [32]. The DFT calculations were based on the Perdew, Burke, and Ernzerhof (PBE) [33] generalized gradient approximation (GGA). Our calculations did not include the effect of spin-orbit coupling due to the absence of the spin-orbit effect for the *s*-block metals. The cutoff energy for the plane wave basis was set to 400 eV. We allowed the system to relax ionic positions, cell volume, and cell shape during structure relaxation until the maximum force on each atom was less than 0.01 eV/Å. Cubic and orthorhombic crystal structures were investigated for all the perovskite compositions. We used 3 × 3 × 3 supercells with 135 atoms in the cubic systems and 2 × 2 × 1 supercells with 80 atoms in the orthorhombic systems. For structural relaxation, we used a 3 × 3 × 3 k-points in the cubic systems, whereas we used a 2 × 2 × 3 k-points in the orthorhombic structures. An 8 × 8 × 8 and 8 × 8 × 12 r-centered k-point grid was used for the density of states (DOS) calculation for the cubic and orthorhombic structures, respectively. VESTA software was used to visualize the charge densities and crystal structures [34]. LOBSTER [35] software was used to extract the bonding information between the atoms using the concept of the crystal orbital overlap population (COOP). We note that for the bonding interactions, the orbital overlap between the orbitals is considered positive, and for the antibonding interaction, the overlap between orbitals is considered negative. Hence, for the antibonding interaction, value of COOP factor is negative.

## 3. Results and Discussion

### 3.1. Atomic Structure and Electronic Properties of Cubic Perovskites

**Figure 1:**
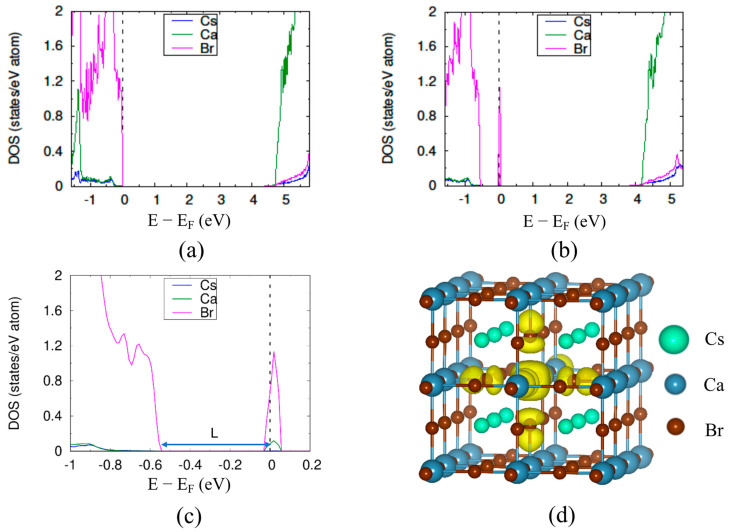
The partial DOS of bulk CsCaBr_3_ (**a**) without defect and (**b**) with the antisite defect. (**c**) magnified portion of the plot (**b**) indicating the ‘*L*’ (eV) value and (**d**) Electron charge density isosurface plot for the energy range −0.9 to 0.1 eV corresponding with (**b**).

We start with the atomic and band structure calculations with cubic CsCaBr_3_, an experimentally observed perovskite, which serves as a representative of the *s*-block ABX_3_ perovskite. It is notable that our calculated lattice parameter and band gap for defect free CsCaBr_3_ was 5.77 Å (see Table S3 in the Supplemental Information) and 4.40 eV (Figure 1a), respectively, which is in good agreement with the experimentally measured lattice constant and band gap of 5.69 Å and 4.39 eV [27], respectively. The VBM consists of Br 4*p* states while the Ca 3*d* orbitals are the major contributor of the CBM. The Cs atom had no significant contribution at VBM or CBM, which is also commonly observed in both *s*-block and *p*-block halide perovskites [27,36]. In the case of CsCaBr_3_ with an antisite defect, a localized electronic defect is observed within the bandgap at the position *L* = 0.56 eV, higher than the VBM (see partial DOS plot in Figure 1b and its magnified version in Figure 1c). To investigate the origin of the localized electronic defect in the CsCaBr_3_ with an antisite defect, we calculated partial electronic charge density at the energy level where the localized electronic defect was formed, as shown by the yellow regions in Figure 1d. The charge density plot shows that that the localized electronic state originated from the columbic interaction between the defect bromine atom at the center and the six surrounding bromine atoms, with the defect bromine atom making the major contribution. Further, the negative value of crystal orbital overlap population (COOP) in our analysis indicates the presence of an antibonding interaction between the 4*p*-4*p* orbitals of the antisite Br atom and the surrounding Br atoms (see Figure S1 in the Supplementary Information). From the molecular orbital theory, we know the energy of antibonding orbitals is higher than the energy of the individual atomic orbital [37]. For this reason, the defect state forms at a higher energy level compared to the VBM. In addition, we found that the bond-length between the defect Br atom and the surrounding Br atoms was 2.4% higher than the initial Ca–Br bond-length in defect-free case (see Table S1 in the Supplemental Information). The bigger size of the Br atom compared to the Ca atom, as well as the repulsion generated by the negative charges of the Br atoms, caused the bond-length to increase.

We hypothesized that the energy level position of the localized defect state created by the antisite defect strongly depends on the antibonding interactions between the *p*-orbitals of halogen atoms and the size of halogen atom. Hence, the position of the localized defect can be predicted based on the intrinsic properties of elements, such as the atomic size of X [38] and the bond dissociation energy (BDE) of B–X [39]. Halogens atomic size reflects the energy of the outer orbital that are taking part in bonding or antibonding interactions. Similarly, a bond’s BDE value provides information about the bond’s strength or the quantitative measure of the orbital interactions between molecular orbitals participating in the bond. In addition, in a perovskite system with a large BDE of the B–X bond, creating an antisite defect will require a greater amount of energy. Therefore, the energy of the electronic state that arises by breaking the B-X bond will surely be a function of the BDE of the B–X bond.

**Figure 2:**
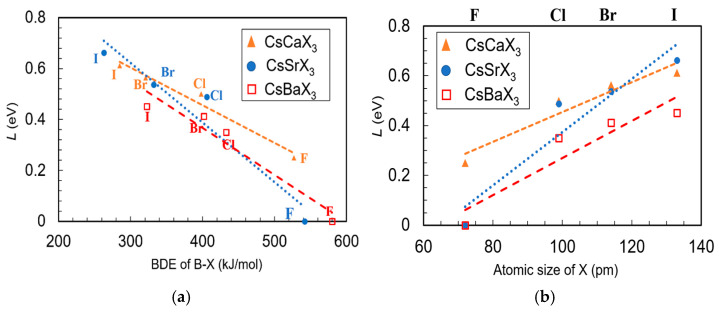
Dependence of cubic CsBX_3_ (B = Ca, Sr and Ba and X = I, Br, Cl and F) defect position with respect to VBM on (**a**) BDE of B-X bond and (**b**) atomic size of X. Dotted lines are for eye-guiding the trend.

To test our hypothesis, we calculated the band structure of a series of cubic ABX_3_ (A = Cs; B = Ca, Sr, or Ba; X = I, Cl, or F) structures with antisite defects. It is important to note that all of the B and X elements we used in our calculations belong to the same group in the periodic table. Therefore, the natures of the VBM and CBM are equivalent to the CsCaBr_3_ structure, i.e., the VBM consists of X n*p* and the CBM is composed of B *nd* orbitals, where *n* is the principal quantum number, and the higher the value of *n*, the higher the energy of the orbital. Based on our hypothesis, we expected that for a fixed B metal with decreasing sizes of halogen atoms from I to F, the position of the localized electronic defect will shift gradually towards the VBM as the energy of the outermost orbital decreases from I(5*p*) > Br(4*p*) > Cl(3*p*) > F(2*p*). For example, the CsCaCl_3_ structure with a smaller sized Cl atom (99 pm) [38] and higher BDE of the Ca–Cl bond (398 kJ/mol) [32] will produce a localized defect closer to the VBM when compared to the CsCaI_3_ structure with a bigger I atom (133 pm) [38] and lower BDE of the Ca–I bond (285 kJ/mol) [39] in presence of an antisite defect. Figure 2a,b show the calculated relative position of the electronic defect state (*L*) as a function of the BDE of the B–X bond and the size of X atom, respectively. The individual density of state calculations for all the cubic ABX_3_ for both pristine and the structures with X_B_ antisite defects are shown in the Supplementary section (see Figures S4–S14 in the Supplemental Information). As theorized earlier, for each B = Ca, Sr, and Ba, the value of *L* decreased gradually from I to F making the localized defect state nearer to the VBM. For all calculations with fluorine (except B = Ca), the defect state merges with the VBM, and we consider *L* as zero. Figure 2 clearly shows that the energy gap *L* is strongly correlated with the BDE of the B–X bond and the size of X. Therefore, it is essential to understand how intrinsic properties are related to the position (*L*) of the localized electronic state.

The higher the BDE of B–X, the stronger the interaction between B and X, as BDE is a measure of the strength of a bond. The formation of the X_B_ antisite defect requires breaking a B–X bond and replacing the B atom with a defect X atom. The defect X atom interacts with the surrounding X atoms to produce the localized defect state as mentioned earlier. For a given X atom, a stronger B–X bond reduces the X atom’s interaction with the defect X atom, and the localized defect state forms at a lower energy level with respect to the VBM. For instance, the Cl(3*p*)–Cl(3*p*) interaction energy will be much lower than that of I(5*p*)–I(5*p*). The BDE and bond-length are directly related to each other. Shorter bonds have a higher BDE and vice versa.

To test our hypothesis, we measured the bond-length between the antisite defect atom and the surrounding halogen atoms and compared them to the Ca–X bond-length for the structure with no defect for the CsCaX_3_ systems. We observed the maximum bond-length increase for CsCaI_3_ (3.5%) and the minimum for CsCaF_3_ (1.3%), which corresponds to the *L* values for these systems (see Table S1 in the Supplemental Information). Again, the localized electronic defect state originated from the n*p*-n*p* antibonding interaction between the halogen atoms. The energy of the interacting *p*-orbitals plays an important role in determining the position of the localized electronic defect state. The energy of the outermost *p*-orbitals followed the order F(2*p*) < Cl(3*p*) < Br(4*p*) < I(5*p*). Therefore, the localized electronic state originated from the I(5*p*)–I(5*p*) antibonding interaction in CsCaI_3_ located at the higher *L* value compared to the position of the electronic defect state due to Cl(3*p*)–Cl(3*p*) interaction in CsCaCl_3_.

**Figure 3:**
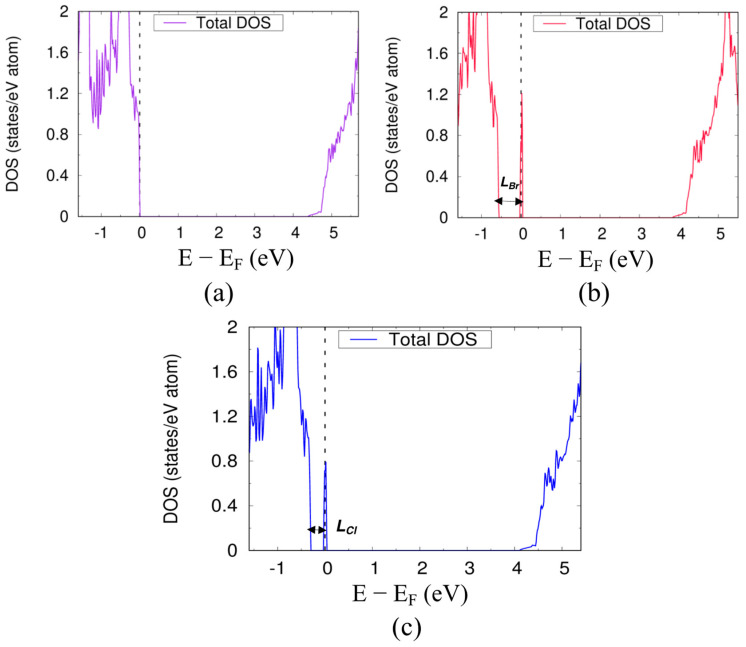
The total DOS of bulk cubic CsCaBr_3_ (**a**) without defect, (**b**) with Br_Ca_ antisite defect (*L_Br_*) and, (**c**) with Cl_Ca_ doping (*L_Cl_*).

To further establish the relationship between the energy of the halogen orbitals and the position of the localized electronic defect state in CsCaBr_3_, we substituted a Cl atom on the Ca atom site (Cl_Ca_), instead of a Br on a Ca site, as it was done for an antisite defect in CsCaBr_3_. We found that the chlorine atom doping creates a localized electronic defect near to the VBM (*L_Cl_ =* 0.34 eV) in comparison to the localized electronic defect state that forms due to Br_Ca_ (*L_Br_* = 0.56 eV) (see Figure 3b,c). The lower energy of Br(4*p*)–Cl(3*p*) antibonding interaction in Cl_Ca_ compared to the Br(4*p*)–Br(4*p*) interaction in the Br_Ca_ antisite defect caused the localized electronic defect to form near to the VBM. Hence, halide perovskites doped with smaller size halogen atoms or a mixed-halide perovskite should show fewer tendencies to form deep trap states.

In summary, for the cubic structures, the position of the localized electronic defect state is a function of the BDE of the B–X bond and the size of the defect X atom. The higher BDE of the B–X bond and the lower size of X create a localized electronic defect closer to VBM. The position of the localized electronic state can be controlled by choosing an appropriated-sized halogen atom.

### 3.2. Atomic Structure and Electronic Properties of Orthorhombic Perovskites

**Figure 4:**
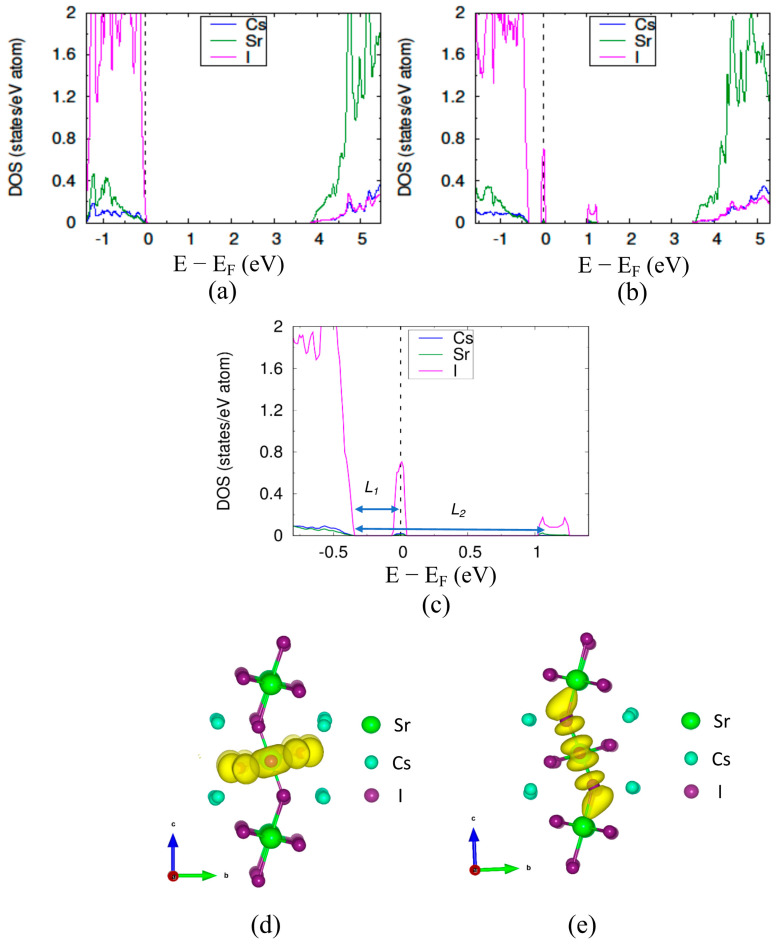
The partial DOS of orthorhombic bulk CsSrI_3_ (**a**) without defect and (**b**) with the antisite defect. (**c**) magnified portion of the plot (**b**) indicating the ‘*L*_1_’ (eV) and ‘*L*_2_’ (eV) value and the electron charge density isosurface plot for the energy range (**d**) −0.9 to 0.1 eV and (**e**) 1 to 1.2 eV corresponding with (**b**).

Depending on the temperature, the perovskites may exhibit structures with less symmetry than the cubic structures. In the case of the highly symmetric cubic structure, the B–X bond distances are isotopic and remain so when an antisite substitution changes the bond distances and results in only one localized electronic state at a singular location within the band structure. However, for a less symmetrical crystal of the same ABX_3_ composition, the B–X bond distances can be anisotropic. In those cases, we hypothesized that, unlike the cubic crystal structure, the presence of an antisite defect in a less symmetrical crystal structure can create electronic defect states at two or more different positions within the band gap. To test this hypothesis, we introduced an antisite defect within orthorhombic CsSrI_3_ (a = 4.81 Å, b = 15.78 Å, and 12.37 Å), a frequently studied *s*-block perovskite structure with a *Cmcm* space group (space group No. 63) [30,40]. This orthorhombic structure differs from the symmetric cubic structure due to (a) a different arrangement of atoms and (b) the tilted octahedra of [SrI_6_] compared to the undistorted octahedral network in a cubic structure. The octahedra consist of one central Sr atom with four equatorial iodine atoms in the same plane (equatorial) with the Sr atom and two apical iodine atoms located out of the plane (see Figures S2 and S3 in the Supplemental Information). The measured Sr–I–Sr bond angles in the orthorhombic structure are 94.5° (equatorial) and 141.8° (apical) as compared to the 180° Ca–Br–Ca bond angle in the cubic CsCaBr_3_. Figure 4a,b represent the electronic DOS of orthorhombic CsSrI_3_ without and with antisite defect, respectively. Our study revealed that, unlike the cubic structures, the antisite defect within orthorhombic structure creates localized electronic defect states at two different energy level positions within the bandgap, which we named defect-1 and defect-2. Defect-1 and defect-2 lie at the energy level *L*_1_ = 0.58 eV and *L*_2_ = 1.6 eV above VBM, respectively. To find the origin of both defect-1 and defect-2, we plotted the charge densities at the energy levels where they are formed as shown in Figure 4d,e, respectively. Similar to the cubic case, the yellow regions depict the electronic charge contributions from all the atoms at that energy level. The charge density plot shows two different kinds of iodine associations which account for the formation of the defect states *L*_1_ and *L*_2_.

**Figure 5:**
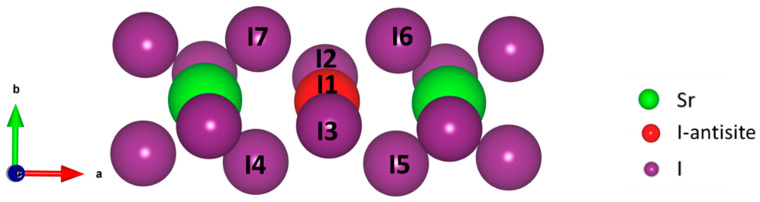
Antisite defect (I1) with all the neighboring atoms at a maximum distance of 4Å. I4, I5, I6, and I7 are in an equatorial plane with the I1. Atoms I2 and I3 located at the apical positions with respect to I1.

Figure 5 shows the two different groups of iodine atoms present in the orthorhombic structure with the defect. I1 is the antisite defect. I4, I5, I6, and I7 are bonded with I1 in the equatorial plane, and the I2, I3, and I1 are bonded along the c-axis (apical). Resembling the cubic structure, the bond-distance between I1 and I4, I5, I6, and I7 (3.81 Å) increased compared to the Sr–I bond-distance (3.33 Å) in the structure with no defect. In contrast, the bond-distance between I1 and I2, I3 (2.94 Å) was lower than the Sr–I bond-distance (3.28 Å) in the defect-free structure. These observations relate to the formation of two different iodine associations and correlate well with the previous discussion of the effect of the I_Pb_ antisite defect on the MAPbI_3_ (001) surface [41].

The Sr-I bond is ionic in nature due to the large electronegativity differences between Sr (EN~0.95) and I (EN~2.66). In the presence of the antisite defect (I_Sr_), the bond between Sr and I breaks at the defect site and the negative charge cloud shifts towards the I atom. These I atoms with an excess negative charge tends to share the charge with the newly introduced I atom by forming covalent bonds. To verify the presence of covalency between the iodine atoms, we performed the Bader charge analysis and measured the distance between the antisite defect and the surrounding I atoms, as shown in Table 1. The Bader charge analysis uses electron density to calculate the electronic charges present on individual atoms [42]. The average charge on the iodine atoms in the structure with no defect was −0.776*e*. In the presence of the antisite defect I1, all of the iodine atoms surrounding the I1 had decreased negative charges, whereas the charge on I1 was +0.193*e*. The positive charge on the I1 and the decrease in charges of the surrounding iodine atoms indicates the sharing of charge and, hence, the presence of covalency between them. The I2 and I3 atoms show the maximum reduction of the negative charge (~48%), and the other four iodine atoms, which are aligned in the equatorial plane with I1, show ~15% reduction. The different measures of charge sharing, or reductions of negative charges are correlated to the two kinds of bond distances between the I1 and the six neighboring iodine atoms. The covalency, or sharing of negative charge, resulted in the shorter bond-length between the I1 and the surrounding six I atoms. The shorter bond-distance of 2.94 Å between I1, I2, and I3 is very close to the bond distances between I atoms in the I_2_ molecule (2.7 Å).

In addition, the distance between I1 and I4, I5, I6, and I7 (3.81 Å) is comparable to the van der Waals bond-length of I_2_ (3.96 Å). The shorter bond-length between the first group of atoms (I1, I2, and I3) compared to the second group of atoms (I1, I3, I4, I5, and I6) is due to the higher extent of sharing the negative charge (greater covalency). We named the first group as trimer and the second group as pentamer. The COOP results show that the defect-1 originated because of the antibonding interaction in the iodine pentamer whereas defect-2 forms because of the antibonding interaction in the iodine trimer (see Supplemental Information). Defect-1 forms near the VBM at a lower energy on the bandgap, and the defect-2 forms at the higher energy level on the bandgap. The different positions of defect-1 and defect-2 can be correlated with the antibonding energy. In addition, the antibonding energy is related to bond-distances between the atoms. It has been reported that shorter metal-halide bonds favor stronger antibonding interactions [43]. For instance, the Sn–Cl bond (2.81 Å) in CsSnCl_3_ has a stronger antibonding interaction energy of 2.469 eV compared to 1.213 eV for the Pb–Cl (2.87 Å) bond in CsPbCl_3_ [36,43]. Therefore, the shorter bond-length in the iodine trimer caused the defect-2 to form at the higher level on the bandgap when compared to defect-1.

**Figure 6:**
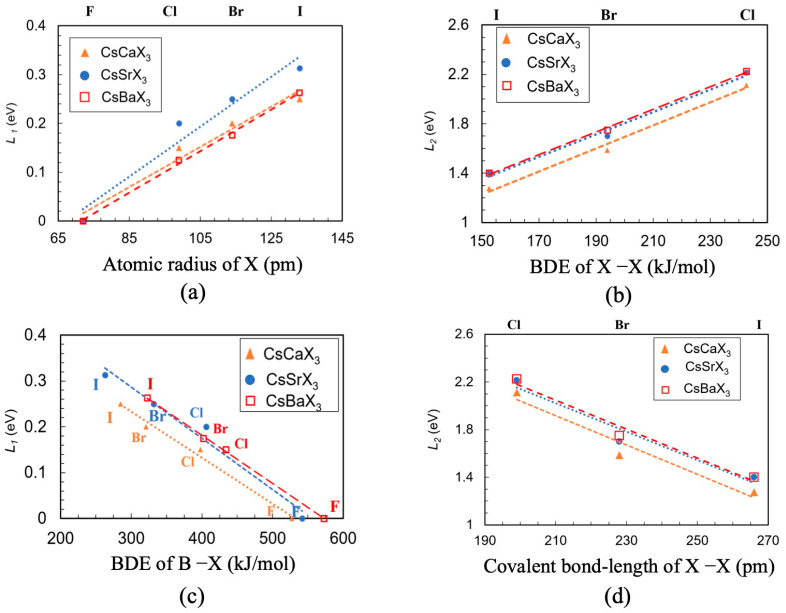
Dependence of orthorhombic CsBX_3_ (B = Ca, Sr and Ba and X = I, Br, Cl and F) defect-1 position (*L*_1_) with respect to VBM on (**a**) BDE of B–X bond and (**b**) atomic size of X and defect-2 position (*L*_2_) on (**c**) BDE of X–X bond and (**d**) covalent bond length of X–X. Dotted lines are for eye-guiding the trend.

From these findings, it is reasonable to link the covalent bond distances of the halogen molecules to the location of defect states within the bandgap. To test this hypothesis, we consider all of the *s*-block orthorhombic ABX_3_ (A = Cs; B = Ca, Sr, and Ba; X = F, Cl, Br, and I) for our band structure and atomic structure calculations (see Figures S15–S25 in the Supplemental Information). As the origin of defect-1 is equivalent to the localized electronic defect in the cubic case, we linked *L*_1_ with the BDE of the B–X bond and the atomic size of X (Figure 6a,b). In Figure 6c,d, *L*_2_ is plotted as a function of two interlinked intrinsic properties, BDE of the X–X bond and the covalent bond-distance of X_2_. For defect-1, we observed the similar dependency of *L*_1_ on BDE of the B-X bond and atomic size of X that we observed in the cubic structure for the parameter *L*, i.e., compounds with lower BDE of B–X and larger atomic sizes correspond to larger values of *L*_1_. Conversely, defect-2 compounds with higher BDE of X–X or smaller covalent bond-distance of X_2,_ show higher values of *L*_2_. For example, CsSrCl_3_ shows a higher *L*_2_ value (2.21 eV) due to a greater BDE of Cl–Cl (436.3 kJ/mol) and a smaller covalent bond-distance of Cl_2_ (198 pm) when compared to the CsSrI_3,_ where the *L*_2_, the BDE of I–I, and the covalent bond-distance of I_2_ is 1.40 eV, 152.2 kJ/mol, and 266 pm, respectively. We were not able to investigate similarly for cases with fluorine, i.e., CsBF_3_ (B = Ca, Sr, and Ba) systems, because the defect-1 merges with the VBM and measuring *L*_1_ i.e., difference of energy from the VBM was not feasible. Our data as presented above shows that the formation and position of the electronic defect states within the band gap in halide perovskites in the presence of an antisite defect are linked to the intrinsic properties of the constituent atoms as well as the crystal structure. Therefore, the energy level where the electronic defects will form in the presence of any other commonly observed point defects, such as cation or anion vacancies, interstitials etc., can be predicted with the basic understanding of the material chemistry and the crystal symmetry. For example, for a B metal vacancy X–X covalent bond length change or the BDE of X–X can play an important role in determining the position of the electronic defect state on the bandgap. Additionally, lower symmetrical crystal structures (e.g., monoclinic or rhombohedral) with two or more different bond distances between B and X will likely produce localized electronic states at multiple energy levels on the bandgap in presence of an antisite defect.

## 4. Conclusions

In summary, we present a DFT-based first-principles study on the electronic structure of ABX_3_ (A = Cs; B = Ca, Sr, and Ba; X = F, Cl, Br, and I) perovskites with cubic and orthorhombic phases in presence of antisite point defect. We observed that the presence of an antisite defect within the cubic perovskite leads to the formation of a localized electronic defect state within the bandgap. The higher BDE of the B–X bond and the lower size of X atom shifts the electronic defect state closer to VBM as much as it merges with VBM in case of CsBaF_3_, a composition with high B–X BDE and smaller sized of halide atom. This implies that the position of the localized electronic state can be controlled by choosing an appropriate cubic perovskite chemistry including mixed halides. Unlike the highly symmetry cubic perovskite structure with 180° B–X–B bond angle, presence of two non-180° B–X–B bond angle leads to the formation of two localized electronic defects in orthorhombic perovskites, a lower symmetric structure. Among the two electronic defects in case of orthorhombic perovskites, interestingly the electronic defect closer to the VBM is linked to the BDE of the B–X bond and the atomic size of X as observed in case of cubic perovskite. However, for the second electronic defect, it was observed that higher BDE of the X–X bond and higher covalent bond-length shift the localized electronic defect away from the VBM band edge. Overall, our calculations provide generic principles to design electronic states of Cs-based perovskites in presence of point defects in terms of readily available intrinsic features of the elements, such as bond-dissociation energy, atomic size, and covalent bond-distance as well as structural characteristic such as B–X–B bond angle. The fundamental principles presented in this research can be extended to screen materials with for a desired electronic structure in wide variety of experimentally observed perovskite crystal structures and point defects.

## Figures and Tables

**Table 1:** Bader charge and distance analysis of defect Iodine and neighboring I atoms for CsSrI_3_ bulk with Sr_I_ defect. I1 is the antisite defect. The average charge on I atoms before introducing defect was −0.776*e*.

Iodine (I)	Charge (e) in the Defect-Free Structure	Charge (e) in Presence of the Defect	Percent Change in Charge (%)	Distance from I1 (Å)
I1		+0.193		
I2	−0.772	−0.394	48.90	2.94
I3	−0.772	−0.394	48.90	2.94
I4	−0.777	−0.660	15.00	3.81
I5	−0.777	−0.660	15.00	3.81
I6	−0.780	−0.660	15.38	3.81
I7	−0.780	−0.660	15.38	3.81

## Data Availability

The data that support the findings of this study are available within the article and its Supplementary Material.

## Supplementary Materials

The following are available online at https://www.mdpi.com/1996-1944/14/4/1032/s1, Figure S1: Crystal orbital overlap population (COOP) analysis for (a) Br_Ca_ antisite defect with surrounding Br atoms in cubic CsCaBr_3_ and (b) I_Sr_ antisite defect with surrounding I atoms in the orthorhombic CsSrI_3_; Table S1: Change in the bond-length of Ca–X (X = F, Cl, Br, I) before and after introducing the X_Ca_ antisite defect; Figure S2: Representation of (a) Cubic (*Pm3m*) and (b) Orthorhombic (*Pnma*) ABX_3_; Figure S3: (a) Axial and (b) Apical bonds in orthorhombic CsSrI_3_; Table S2: The optimized lattice parameter (Å) of cubic ABX_3_ (A = Cs, M = Ca, Sr, Ba and X = F, Cl, Br, I); Table S3: The optimized lattice parameter (Å) of orthorhombic ABX_3_ (A = Cs, M = Ca, Sr, Ba and X = F, Cl, Br, I); Figure S4: The partial DOS of bulk cubic CsCaI_3_ (a) without defect and (b) with antisite defect; Figure S5: The partial DOS of bulk cubic CsCaCl_3_ (a) without defect and (b) with antisite defect; Figure S6: The partial DOS of bulk cubic CsCaF_3_ (a) without defect and (b) with antisite defect; Figure S7: The partial DOS of bulk cubic CsSrI_3_ (a) without defect and (b) with antisite defect; Figure S8: The partial DOS of bulk cubic CsSrBr_3_ (a) without defect and (b) with antisite defect; Figure S9: The partial DOS of bulk cubic CsSrCl_3_ (a) without defect and (b) with antisite defect; Figure S10: The partial DOS of bulk cubic CsSrF_3_ (a) without defect and (b) with antisite defect; Figure S11: The partial DOS of bulk cubic CsBaI_3_ (a) without defect and (b) with antisite defect; Figure S12: The partial DOS of bulk cubic CsBaCl_3_ (a) without defect and (b) with antisite defect; Figure S13: The partial DOS of bulk cubic CsBaBr_3_ (a) without defect and (b) with antisite defect; Figure S14: The partial DOS of bulk cubic CsBaF_3_ (a) without defect and (b) with antisite defect; Figure S15: The partial DOS of bulk orthorhombic CsCaI_3_ (a) without defect and (b) with antisite defect; Figure S16: The partial DOS of bulk orthorhombic CsCaBr_3_ (a) without defect and (b) with antisite defect; Figure S17: The partial DOS of bulk orthorhombic CsCaCl_3_ (a) without defect and (b) with antisite defect; Figure S18: The partial DOS of bulk orthorhombic CsCaF_3_ (a) without defect and (b) with antisite defect; Figure S19: The partial DOS of bulk orthorhombic CsSrBr_3_ (a) without defect and (b) with antisite defect; Figure S20: The partial DOS of bulk orthorhombic CsSrCl_3_ (a) without defect and (b) with antisite defect; Figure S21: The partial DOS of bulk orthorhombic CsSrF_3_ (a) without defect and (b) with antisite defect; Figure S22: The partial DOS of bulk orthorhombic CsBaI_3_ (a) without defect and (b) with antisite defect; Figure S23: The partial DOS of bulk orthorhombic CsBaBr_3_ (a) without defect and (b) with antisite defect; Figure S24: The partial DOS of bulk orthorhombic CsBaCl_3_ (a) without defect and (b) with antisite defect; Figure S25: The partial DOS of bulk orthorhombic CsBaF_3_ (a) without defect and (b) with antisite defect.
