# Supplementary material for: Role of Chemistry and Crystal Structure on the Electronic Defect States in Cs-Based Halide Perovskites
Source: Materials (Basel). 2021 Feb 22;14(4):1032. doi: 10.3390/ma14041032 (PMC7926727; doi:10.3390/ma14041032)
Supplement: Supplementary file 1 [file materials-14-01032-s001.zip › materials-1051700-supplementary.pdf]

materials

MDPI

Supplementary Materials

Role of Chemistry and Crystal Structure on the Electronic Defect States in Cs-Based Halide Perovskites

Anirban Naskar, Rabi Khanal and Samrat Choudhury *

Materials Science and Engineering Program, University of Idaho, Moscow, ID 83844, USA; nask9705@vandals.uidaho.edu (A.N.); rkhanal@uidaho.edu (R.K.)
* Correspondence: samrat@uidaho.edu

1. LOBSTER COOP Plot for Cubic $CsCaBr_3$ and Orthorhombic $CsSrI_3$ in Presence of Antisite Defect

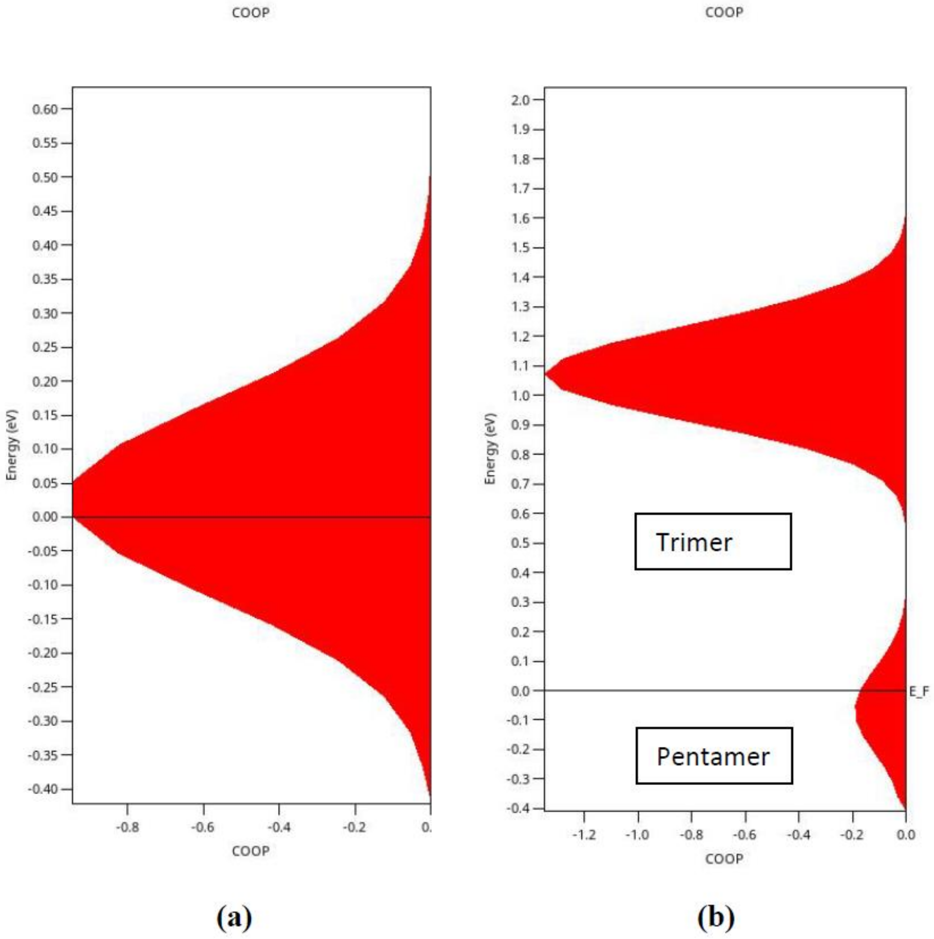

Figure S1. Crystal orbital overlap population (COOP) analysis for (**a**) BrCa antisite defect with surrounding Br atoms in cubic $CsCaBr_3$ and (**b**) ISr antisite defect with surrounding I atoms in the orthorhombic $CsSrI_3$.

Publisher's Note: MDPI stays neutral with regard to jurisdictional claims in published maps and institutional affiliations.

Copyright: © 2021 by the authors. Licensee MDPI, Basel, Switzerland. This article is an open access article distributed under the terms and conditions of the Creative Commons Attribution (CC BY) license (http://creativecommons.org/licenses/by/4.0/).

2. Table Representation Bond-Length Change for Cubic $CsCaX_3$ (X = F, Cl, Br and I)

Table S1. Change in the Bond-length of Ca–X (X = F, Cl, Br, I) before and after introducing the XCa antisite defect.

Systems	Ca–X Bond-Length in Pure Structure (Å)	X_{Ca}–X bond-Length after Introducing the Defect (Å)	Percent Change in Bond-Length (%)
$CsCaI_3$	3.11	3.22	3.5
$CsCaBr_3$	2.88	2.95	2.4
$CsCaCl_3$	2.73	2.78	1.8
$CsCaF_3$	2.24	2.27	1.3

3. Cubic (Pm3m) and Orthorohmbic (Pnma) ABX_3 representation

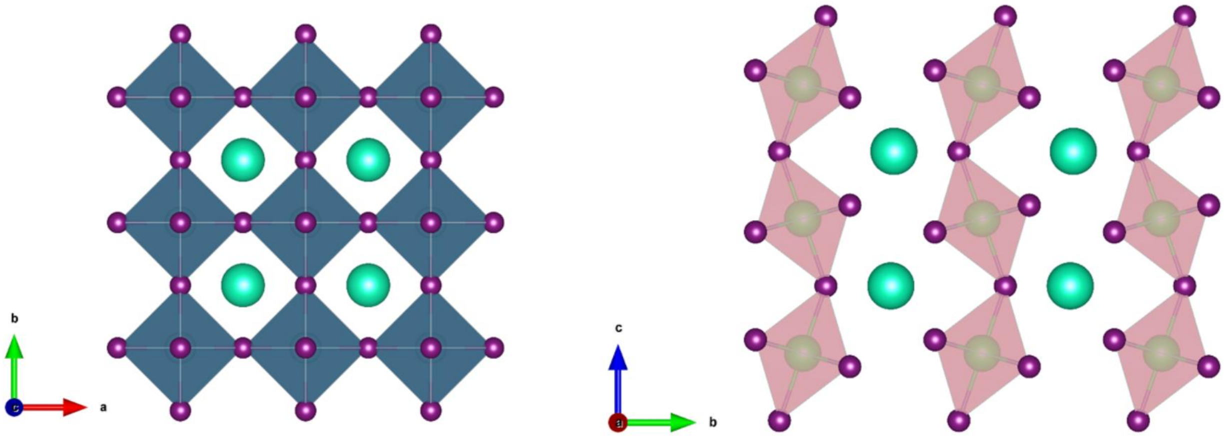

(a) (b)

Figure S2. Representation of (**a**) Cubic (Pm3m) and (**b**) Orthorohmbic (Pnma) ABX_3.

4. Axial and Apical Positions (Angles) in Orthorhombic $CsSrI_3$

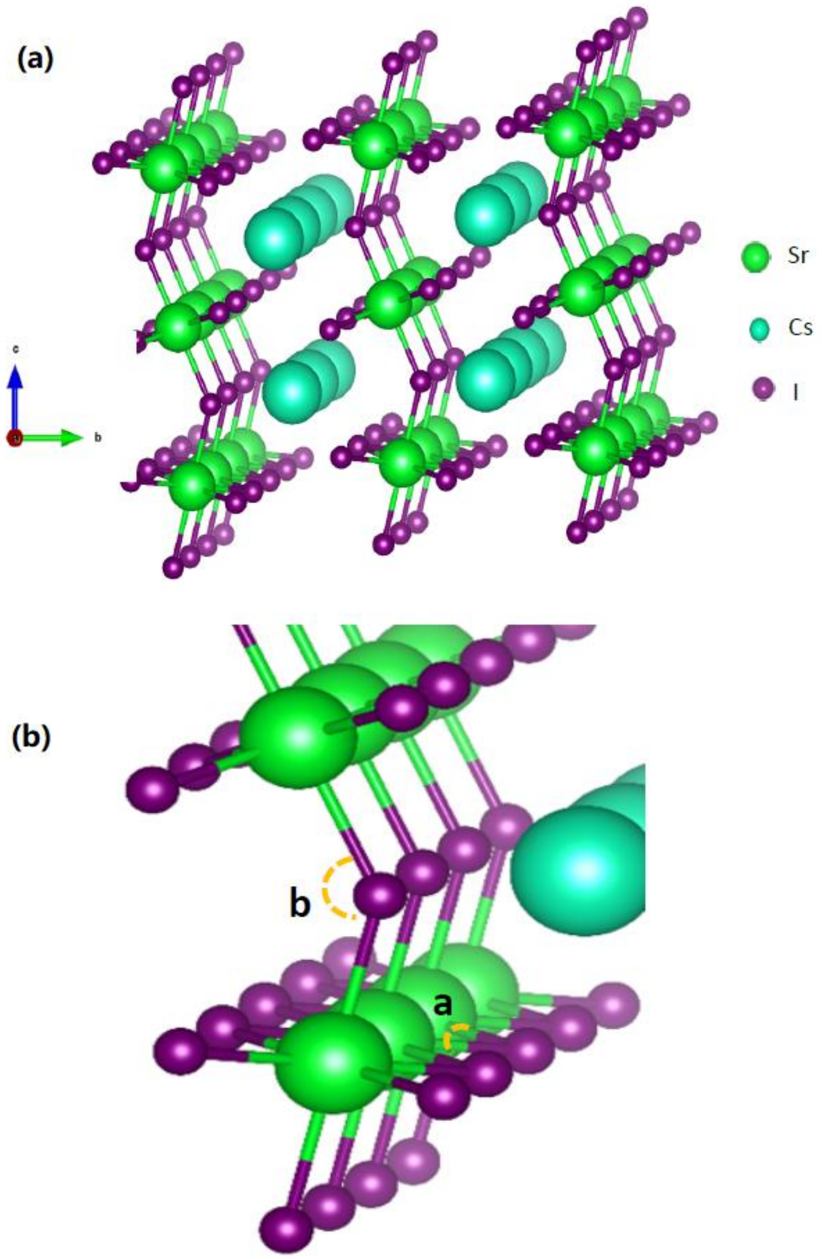

Figure S3. (**a**) Axial and (**b**) Apical bonds in orthorhombic $CsSrI_3$.

5. Computed Lattice Parameters of ABX_3 (A = Cs, M = Ca, Sr, Ba and X = F, Cl, Br, I)

Table S2. The optimized lattice parameter (Å) of cubic ABX_3 (A = Cs, M = Ca, Sr, Ba and X = F, Cl, Br, I).

Systems	a	b	c
$CsCaF_3$	4.58	4.58	4.58
$CsCaCl_3$	5.46	5.46	5.46
$CsCaBr_3$	5.77	5.77	5.77
$CsCaI_3$	6.21	6.21	6.21
$CsSrF_3$	4.82	4.82	4.82
$CsSrCl_3$	5.74	5.74	5.74
$CsSrBr_3$	6.05	6.05	6.05
$CsSrI_3$	6.47	6.47	6.47
$CsBaF_3$	5.15	5.15	5.15
$CsBaCl_3$	6.07	6.07	6.07
$CsBaBr_3$	6.34	6.34	6.34
$CsBaI_3$	6.82	6.82	6.82

Table S3. The optimized lattice parameter (Å) of orthorhombic ABX_3 (A = Cs, M = Ca, Sr, Ba and X = F, Cl, Br, I).

Systems	a	b	c
$CsCaF_3$	3.67	12.50	8.68
$CsCaCl_3$	4.26	14.59	10.51
$CsCaBr_3$	4.50	15.14	11.14
$CsCaI_3$	4.85	16.07	12.01
$CsSrF_3$	3.84	12.79	9.09
$CsSrCl_3$	4.44	14.84	10.90
$CsSrBr_3$	4.66	15.39	11.52
$CsSrI_3$	5.01	16.29	12.39
$CsBaF_3$	4.05	13.28	9.49
$CsBaCl_3$	4.64	15.28	11.26
$CsBaBr_3$	4.85	15.88	11.87
$CsBaI_3$	5.18	16.53	12.84

6. Electronic Band Structures of ABX_3 with and without Antisite Defect

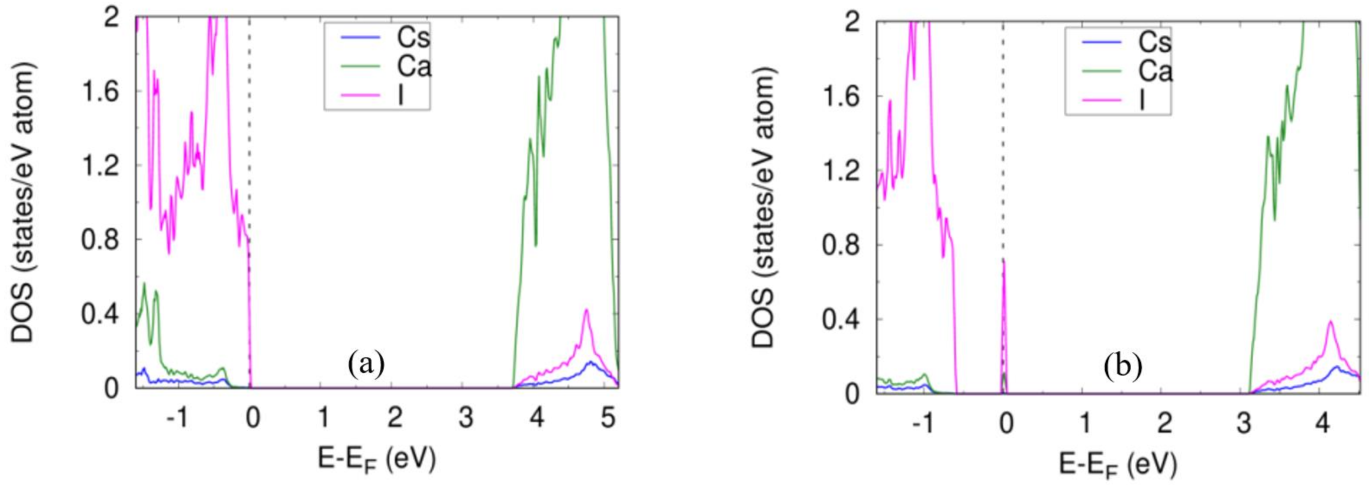

Figure S4. The partial DOS of bulk cubic $CsCaI_3$ (**a**) without defect and (**b**) with antisite defect.

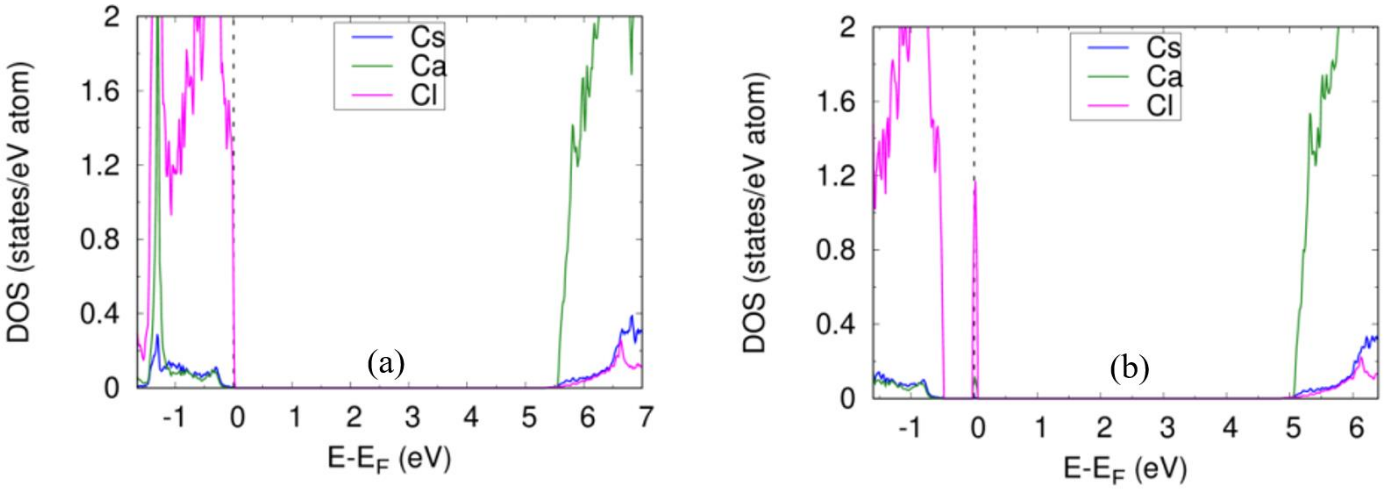

Figure S5. The partial DOS of bulk cubic $CsCaCl_3$ (**a**) without defect and (**b**) with antisite defect.

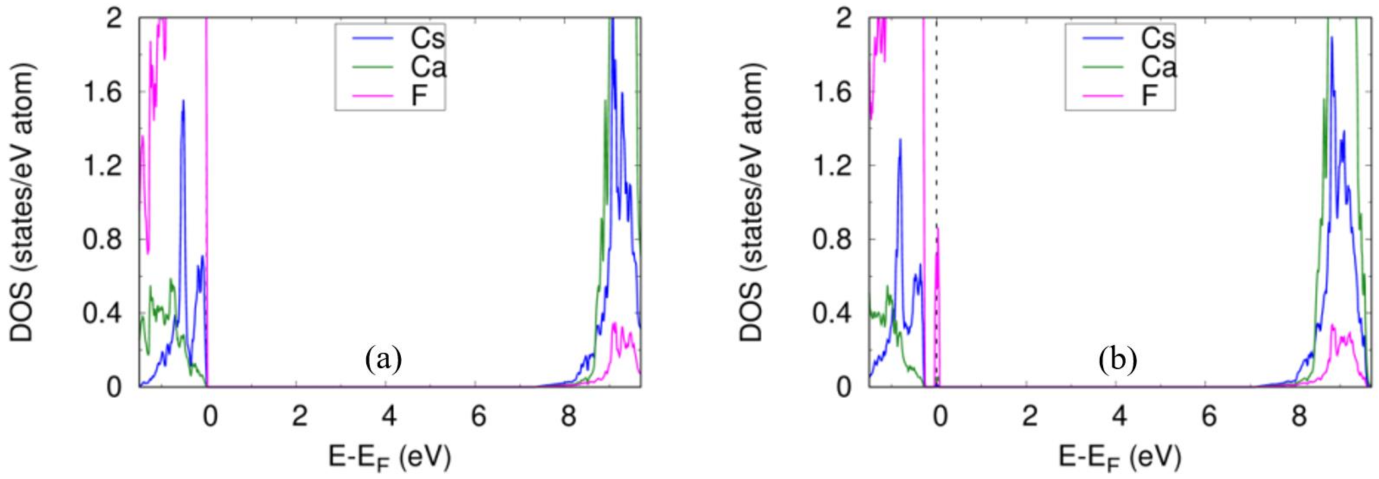

Figure S6. The partial DOS of bulk cubic $CsCaF_3$ (**a**) without defect and (**b**) with antisite defect.

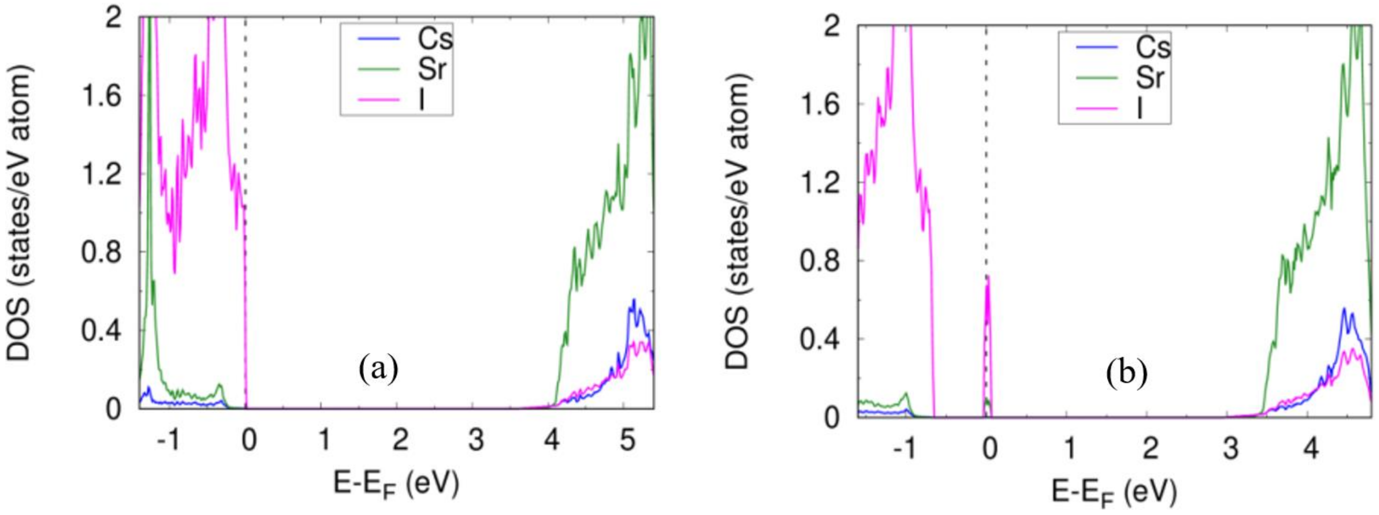

Figure S7. The partial DOS of bulk cubic $CsSrI_3$ (**a**) without defect and (**b**) with antisite defect.

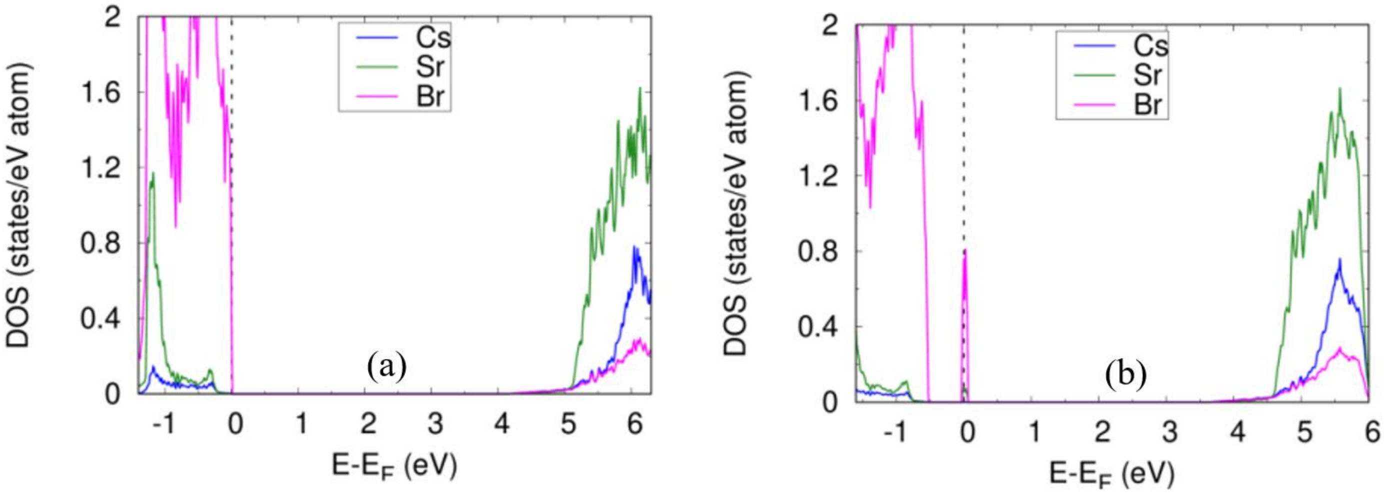

Figure S8. The partial DOS of bulk cubic $CsSrBr_3$ (**a**) without defect and (**b**) with antisite defect.

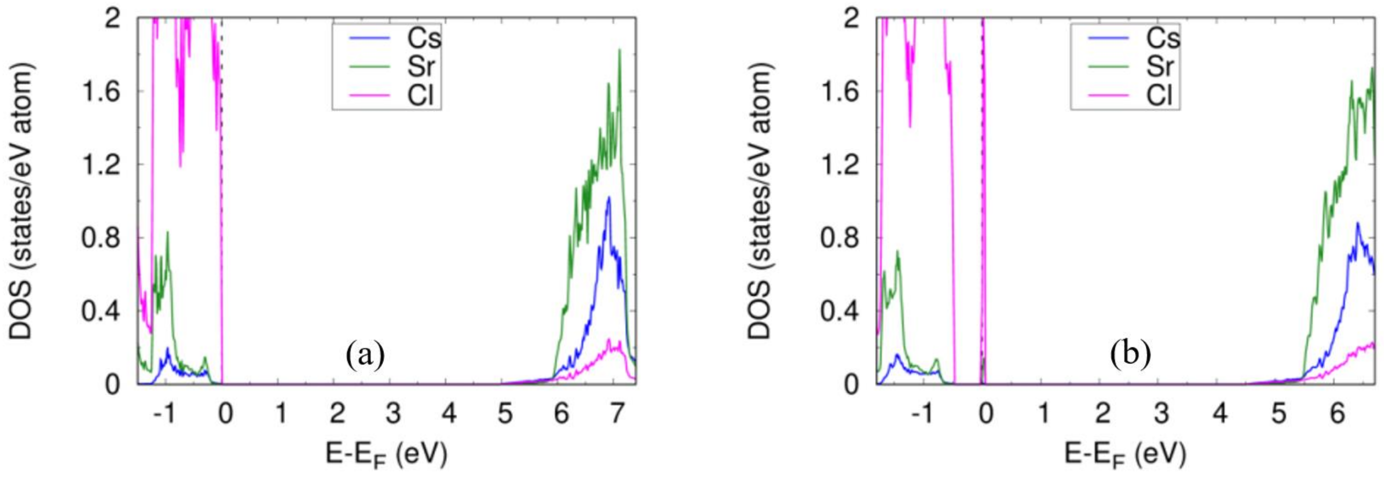

Figure S9. The partial DOS of bulk cubic $CsSrCl_3$ (**a**) without defect and (**b**) with antisite defect.

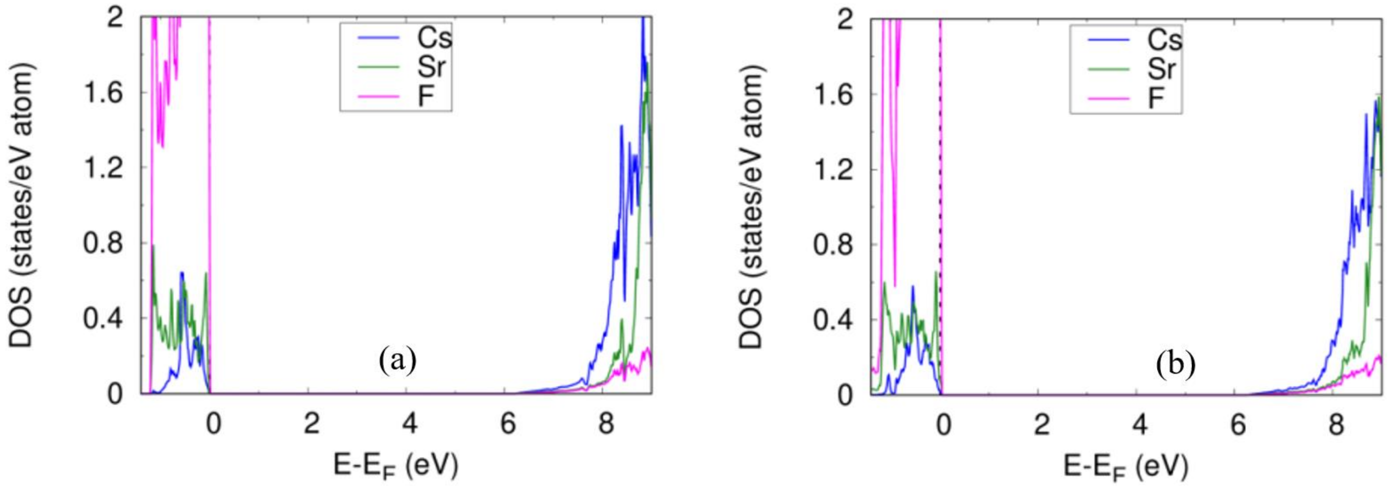

Figure S10. The partial DOS of bulk cubic $CsSrF_3$ (**a**) without defect and (**b**) with antisite defect.

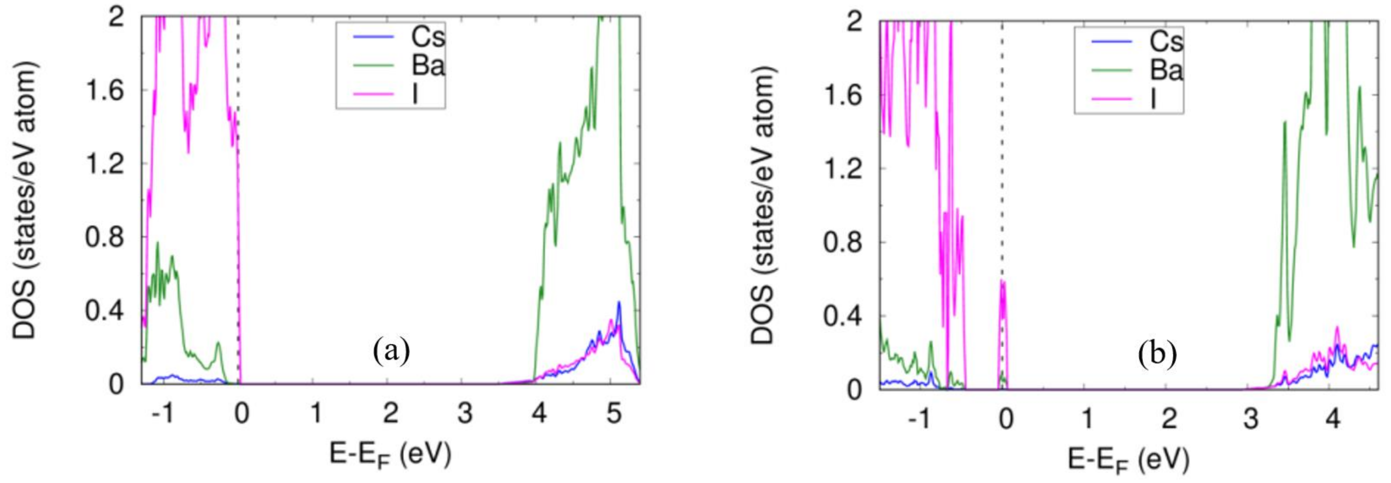

Figure S11. The partial DOS of bulk cubic $CsBaI_3$ (**a**) without defect and (**b**) with antisite defect.

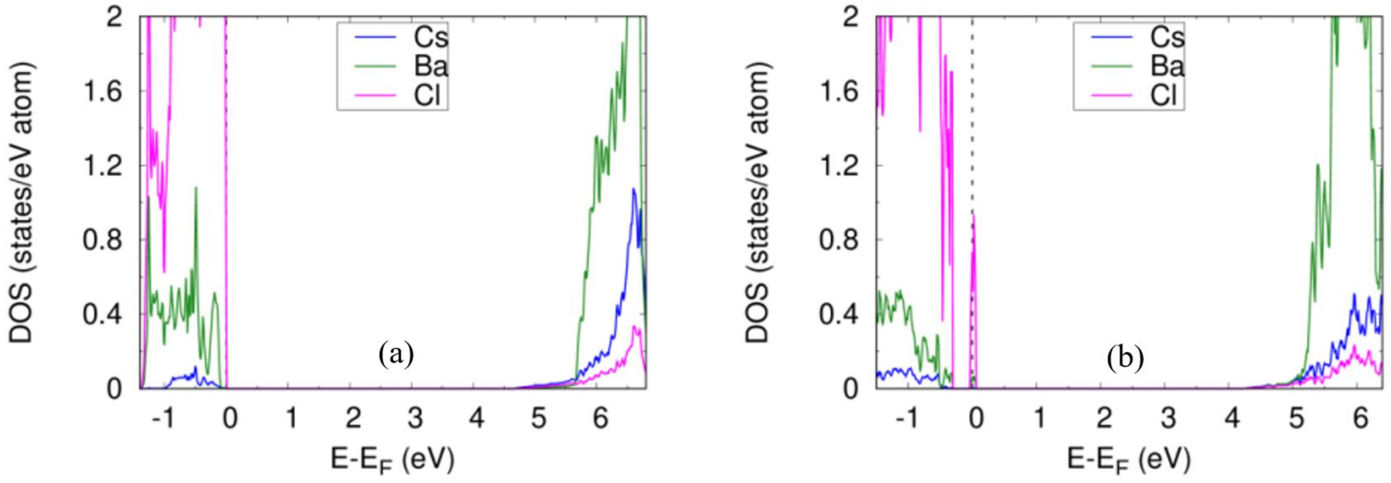

Figure S12. The partial DOS of bulk cubic $CsBaCl_3$ (**a**) without defect and (**b**) with antisite defect.

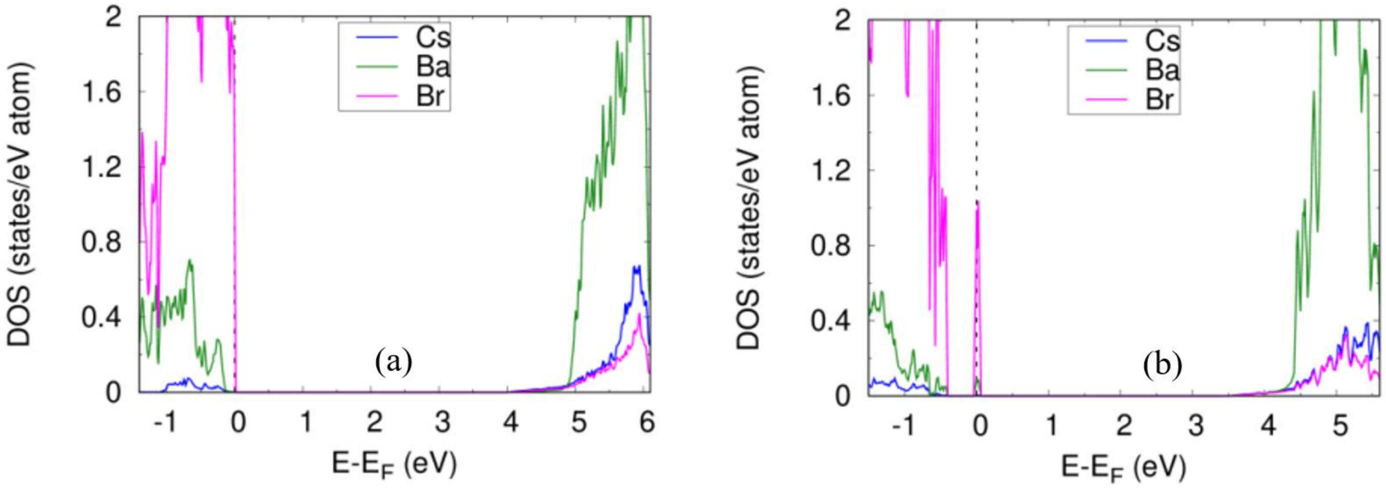

Figure S13. The partial DOS of bulk cubic $CsBaBr_3$ (**a**) without defect and (**b**) with antisite defect.

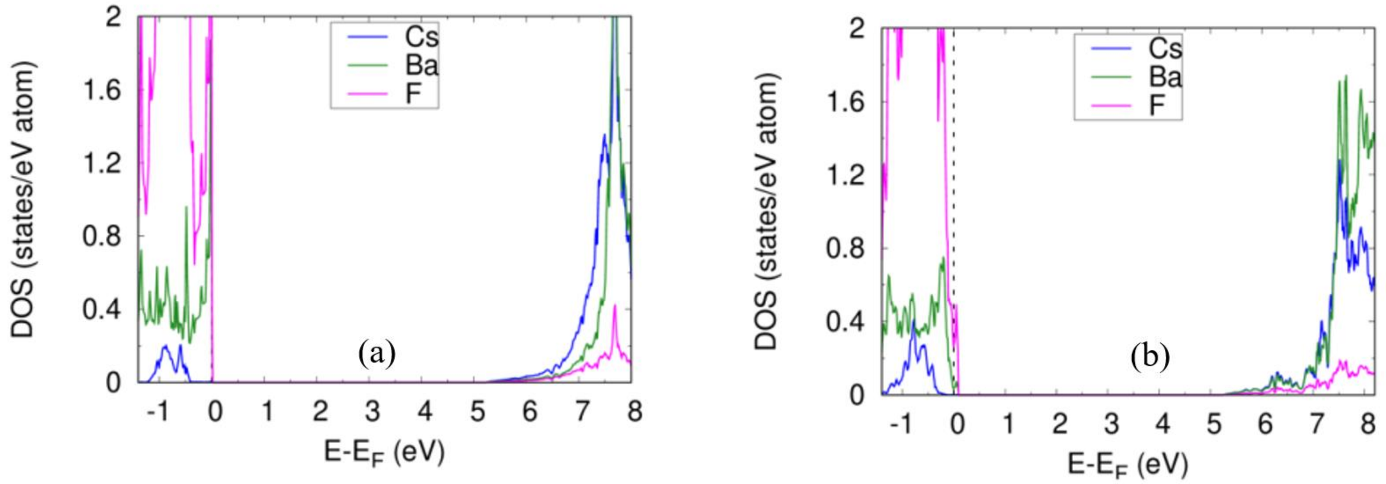

Figure S14. The partial DOS of bulk cubic $CsBaF_3$ (**a**) without defect and (**b**) with antisite defect.

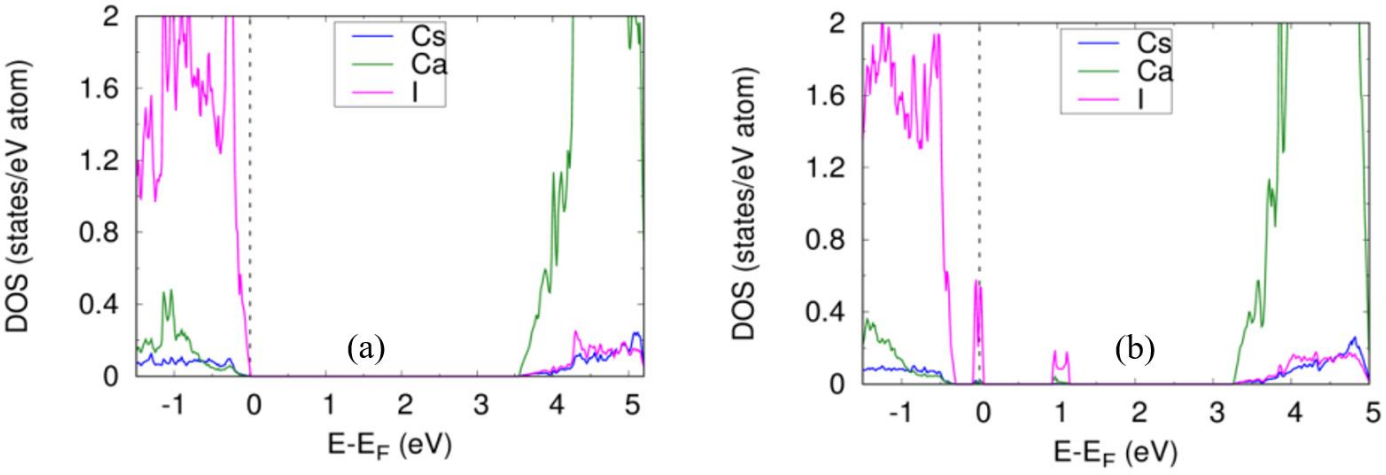

Figure S15. The partial DOS of bulk orthorhombic $CsCaI_3$ (**a**) without defect and (**b**) with antisite defect.

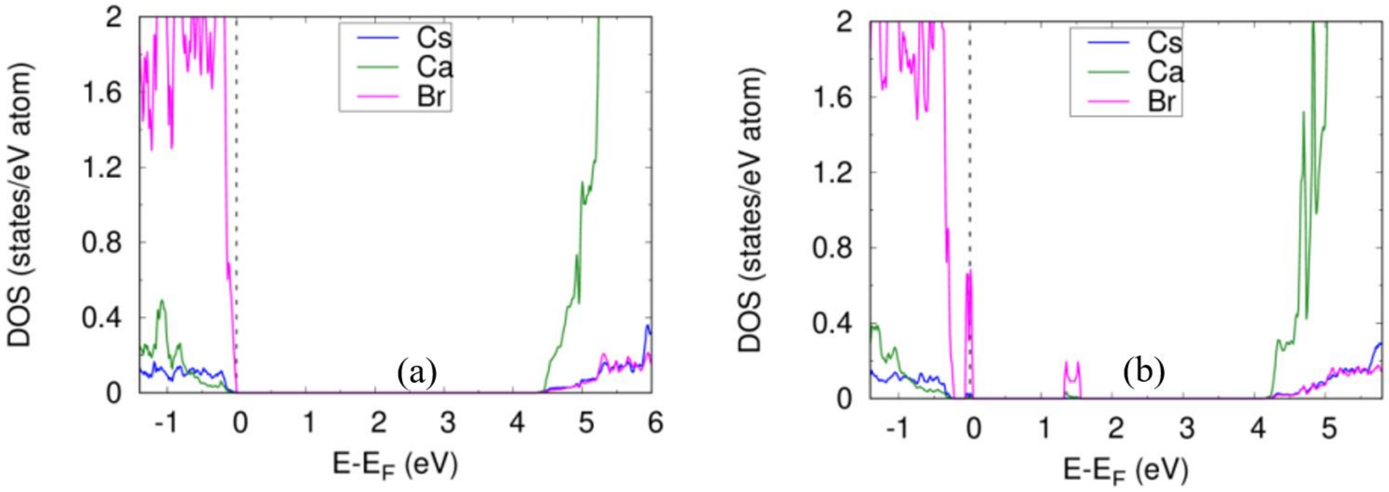

Figure S16. The partial DOS of bulk orthorhombic $CsCaBr_3$ (**a**) without defect and (**b**) with antisite defect.

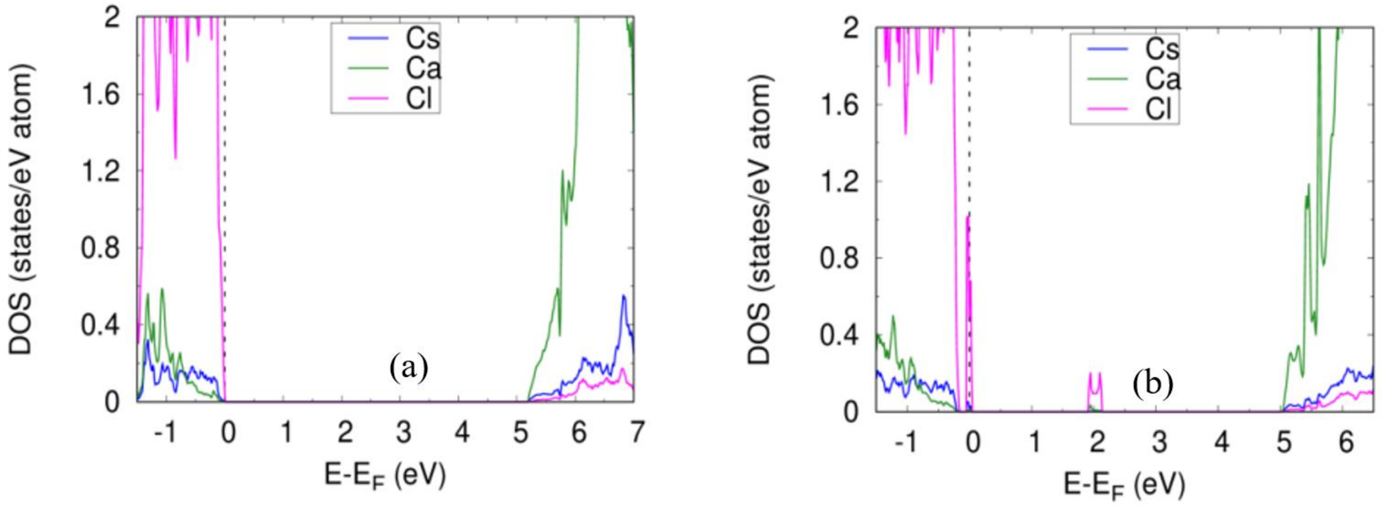

Figure S17. The partial DOS of bulk orthorhombic $CsCaCl_3$ (**a**) without defect and (**b**) with antisite defect.

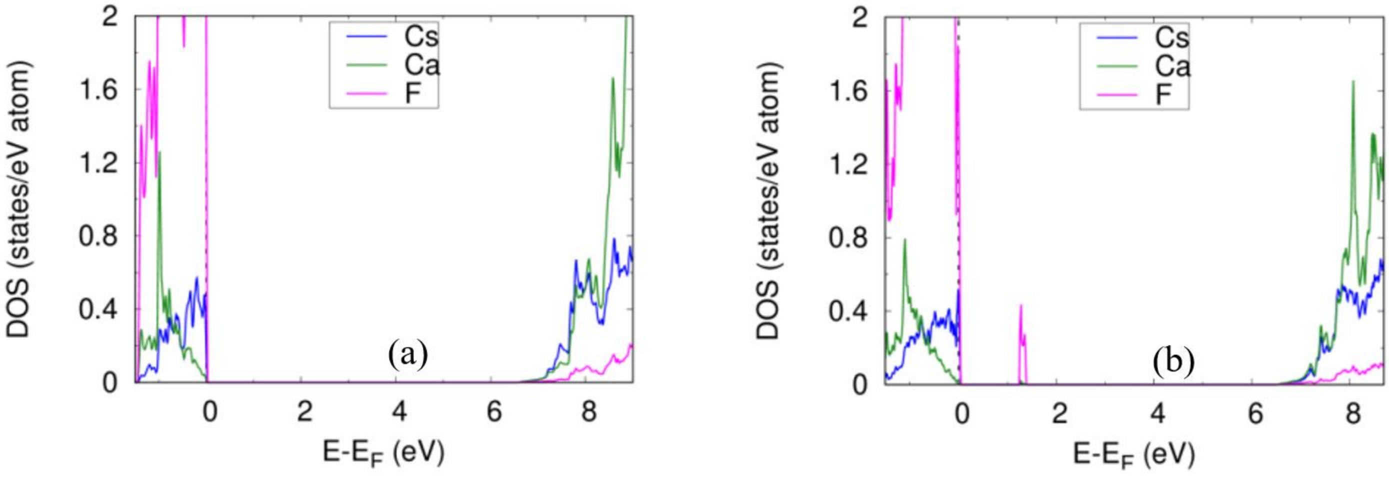

Figure S18. The partial DOS of bulk orthorhombic $CsCaF_3$ (**a**) without defect and (**b**) with antisite defect.

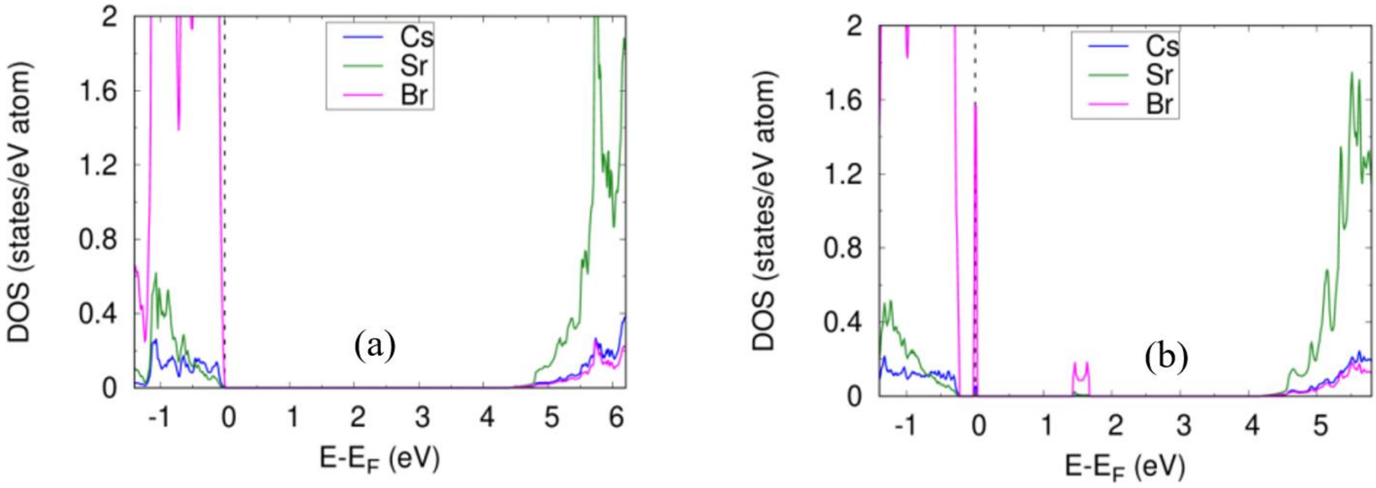

Figure S19. The partial DOS of bulk orthorhombic $CsSrBr_3$ (**a**) without defect and (**b**) with antisite defect.

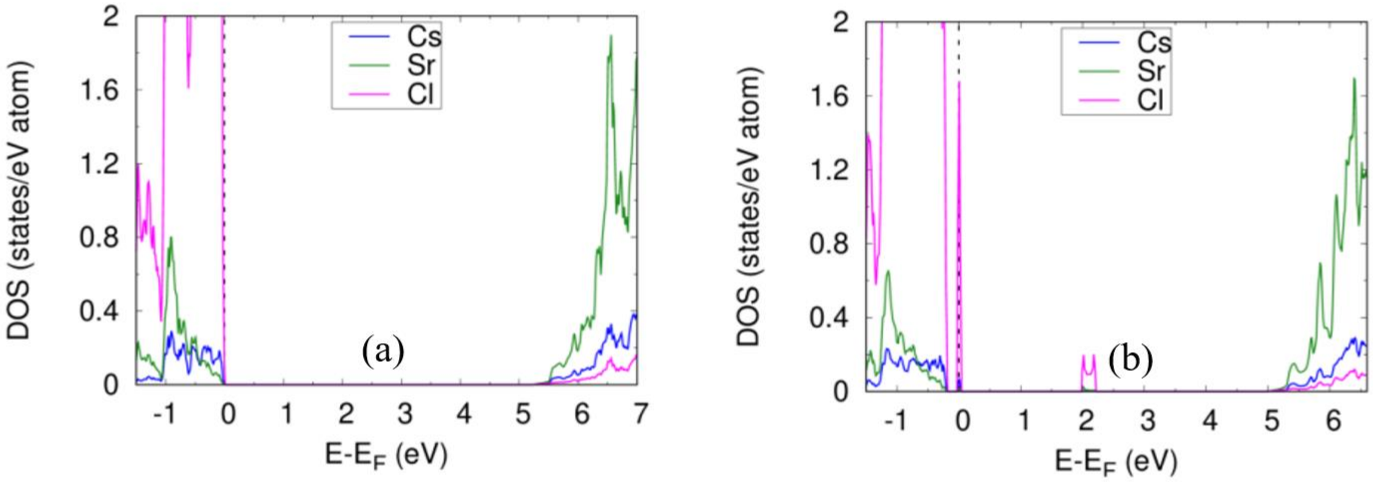

Figure S20. The partial DOS of bulk orthorhombic $CsSrCl_3$ (**a**) without defect and (**b**) with antisite defect.

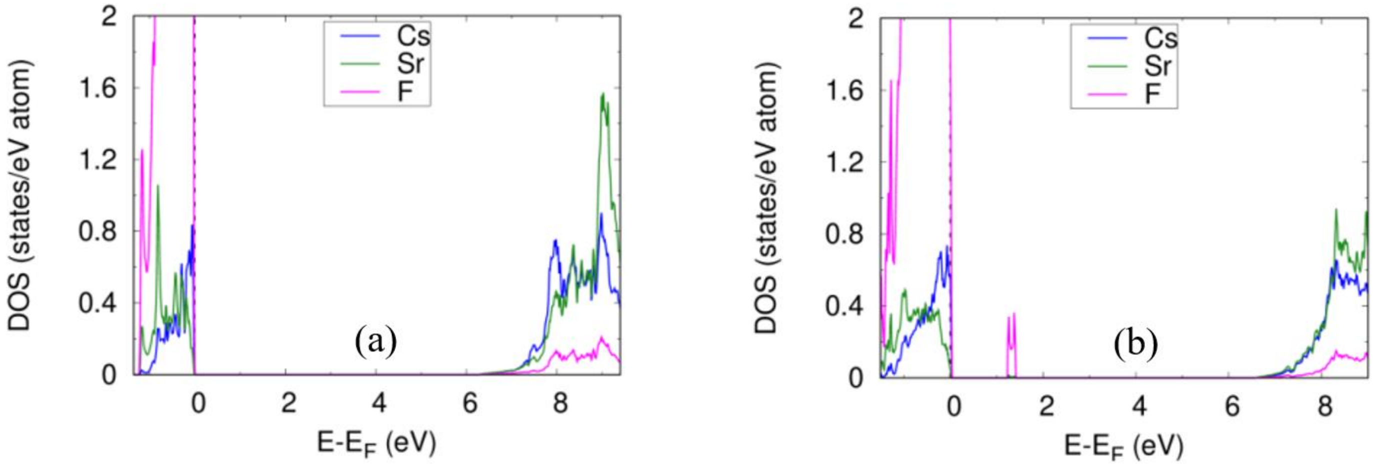

Figure S21. The partial DOS of bulk orthorhombic $CsSrF_3$ (**a**) without defect and (**b**) with antisite defect.

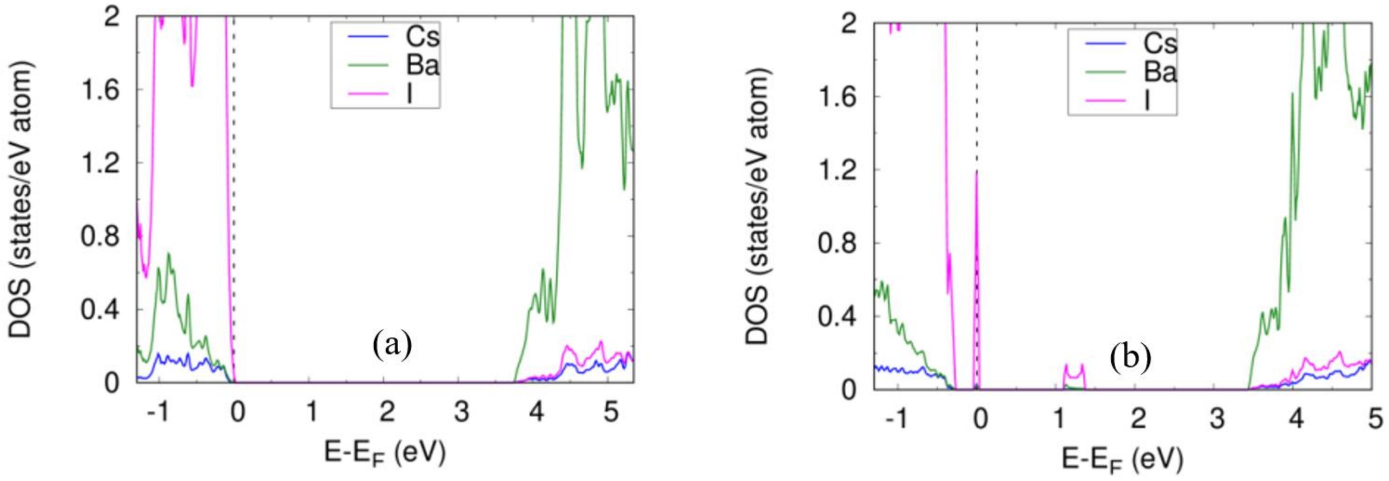

Figure S22. The partial DOS of bulk orthorhombic $CsBaI_3$ (**a**) without defect and (**b**) with antisite defect.

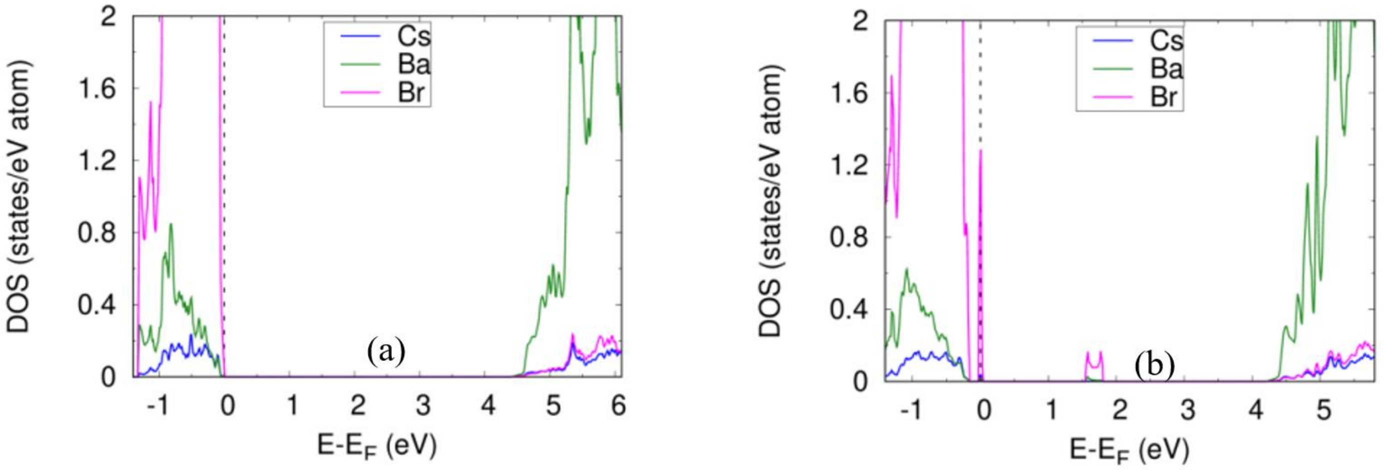

Figure S23. The partial DOS of bulk orthorhombic $CsBaBr_3$ (**a**) without defect and (**b**) with antisite defect.

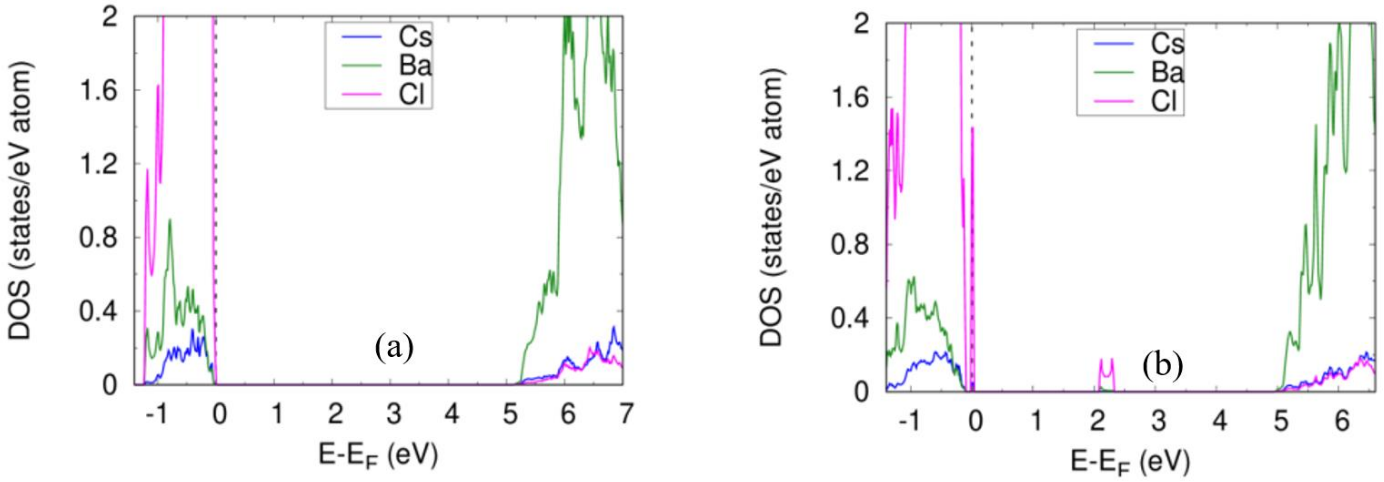

Figure S24. The partial DOS of bulk orthorhombic $CsBaCl_3$ (**a**) without defect and (**b**) with antisite defect.

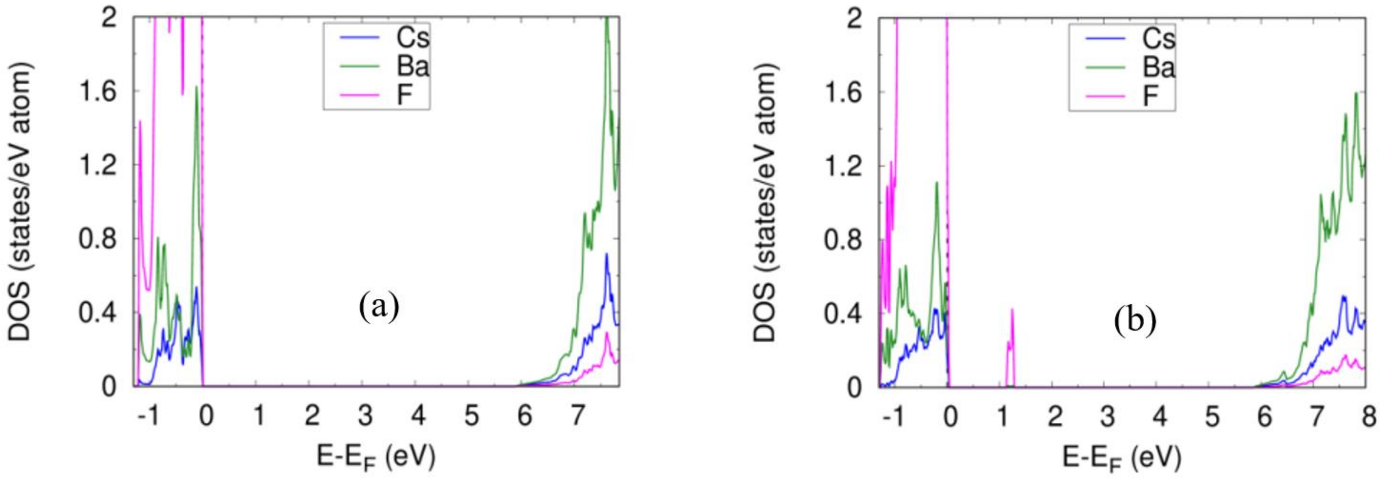

Figure S25. The partial DOS of bulk orthorhombic $CsBaF_3$ (**a**) without defect and (**b**) with antisite defect.
